# Supplementary material for: Photoluminescent Gold/BSA Nanoclusters (AuNC@BSA) as Sensors for Red-Fluorescence Detection of Mycotoxins
Source: Materials (Basel). 2022 Nov 27;15(23):8448. doi: 10.3390/ma15238448 (PMC9740986; doi:10.3390/ma15238448)
Supplement: Supplementary file 1 [file materials-15-08448-s001.zip › materials-1999992-supplementary.pdf]

materials

Supporting information

Photoluminescent Gold/BSA Nanoclusters (AuNC@BSA) as Sensors for Red-Fluorescence Detection of Mycotoxins

Ivana Fabijanić [1], Marta Jurković [1], Daniela Jakšić [2] and Ivo Piantanida [1,*]

Figure S1. Size distribution of AuNC@BSA.

Figure S2. UV/Vis spectrum and excitation spectrum of AuNC@BSA.

Table S1. Lifetime data obtained by measuring lifetime decay of AuNC@BSA at 273 K at different pH **values.**

Figure S3. Fluorescent emission data of HSA upon titration with STC.

Figure S4. Fluorescent emission data of BSA upon titration with FB_1.

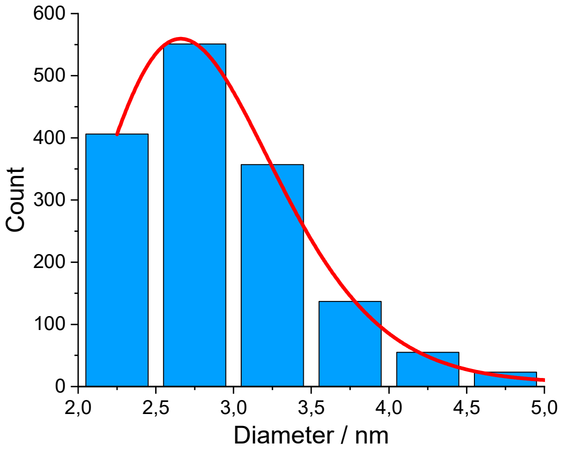

Figure S1. Size distribution of AuNC@BSA. The average nanocluster diameter measured from TEM images using ImageJ software [1] is 2.8 ± 0.2 nm.

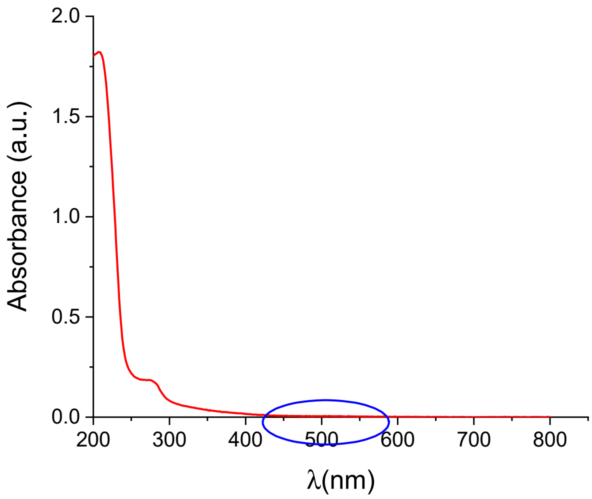

A

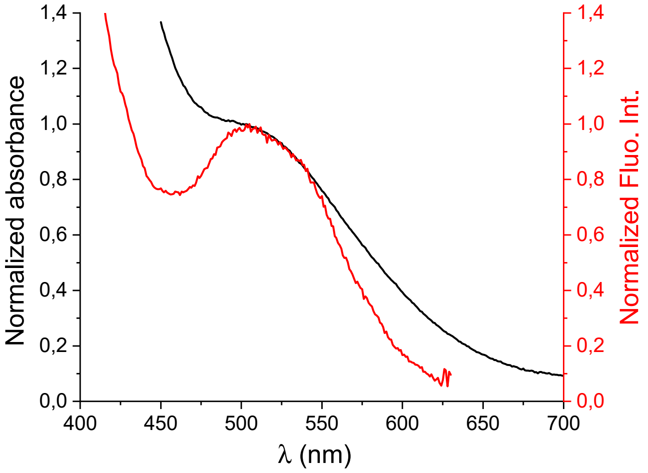

B

Figure S2. A) UV/Vis spectrum of AuNC@BSA in water; **B)** Enlarged area of A) marked by blue circle: UV/Vis spectrum (**black** line) and excitation spectrum (**red** line - λ_{em}= 680 nm) of AuNC@BSA in water.

Table S1. Lifetime data obtained by measuring lifetime decay of **AuNC@BSA at 273 K**.

	**AuNC@BSA (EPLED340)/ns**						**AuNC@BSA** (EPLED560)/ns**			
	**pH = 7.3***		**pH = 10.3**		**pH = 12**		**pH = 10.3**		**pH = 12**	
	**τ**	**Rel/%**	**τ**	**Rel/%**	**τ**	**Rel/%**	**τ**	**Rel/%**	**T**	**Rel/%**
τ_1	24.30 (±10.18)	0.42	169.8 (±31.74)	2.24	168.1 (±25.99)	2.81	8.569 (±1.982)	0.24	54.68 (± 9.0141)	0.85
τ_2	607.3 (±45.21)	15.86	877.9 (±100.3)	33.47	833.4 (±77.03)	32.16	366.5 (±26.51)	8.63	579.497 (±62.86)	19.97
τ_3	1849 (± 36.88)	83.72	1891 (±122.8)	64.29	1812 (±73.36)	65.03	1446 (±27.03)	91.13	1637 (±109.9)	79.18
χ^2	1.0001		0.9914		0.9892		1.0070		1.0502	

* 4 µL of stock solution of AuNC@BSA (pH = 12) in 2 mL of ultrapure water has pH = 7.3. The BSA concentration in a cuvette is 7.15 × 10^{-7} M.

** measurements done with the stock solution (c(BSA) = 3.58·10^{-4} M or c (Au_{25}, AuNC@BSA = 190 µM).

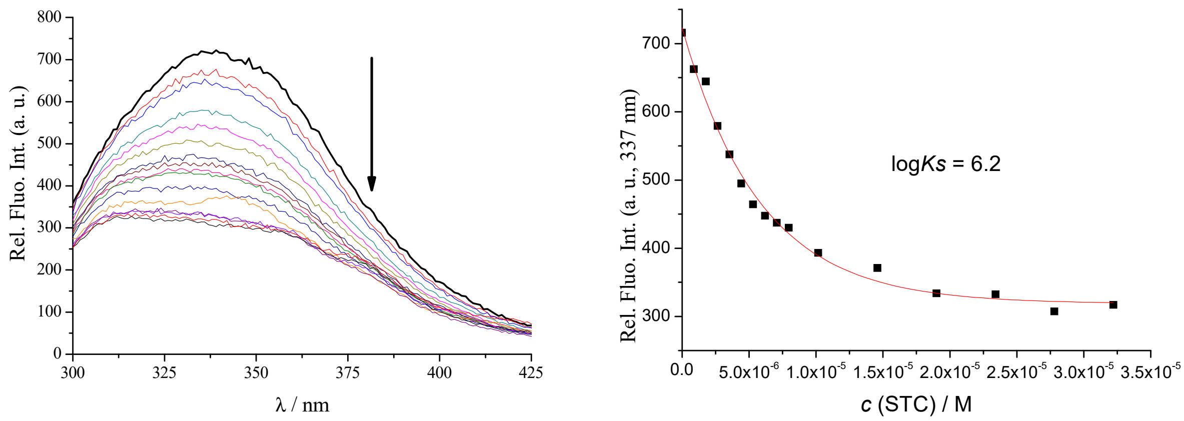

A) B)

Figure S3. A) Changes in HSA emission spectra ($c = 1 \times 10^{-6}$ M, $\lambda_{exc} = 280$ nm) upon titration with STC ($c_{stock} = 9 \times 10^{-3}$ M, in MeCN). B) emission changes at $\lambda = 337$ nm fitted to the 1:1 stoichiometry complex (−). Performed at pH 7.0, Na-cacodylate buffer, $I = 0.05$ M.

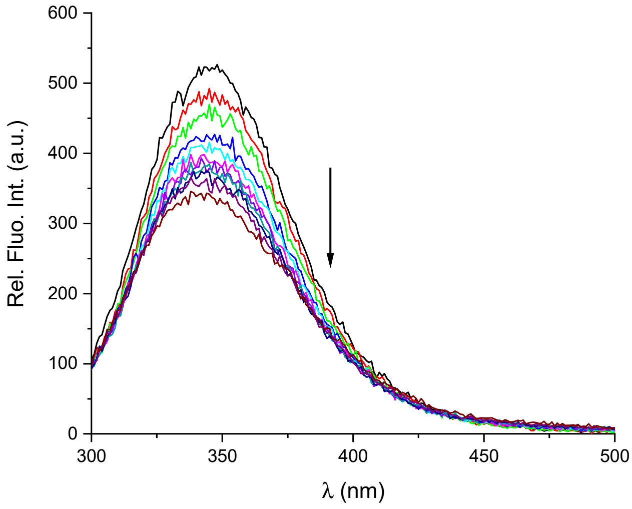

A)

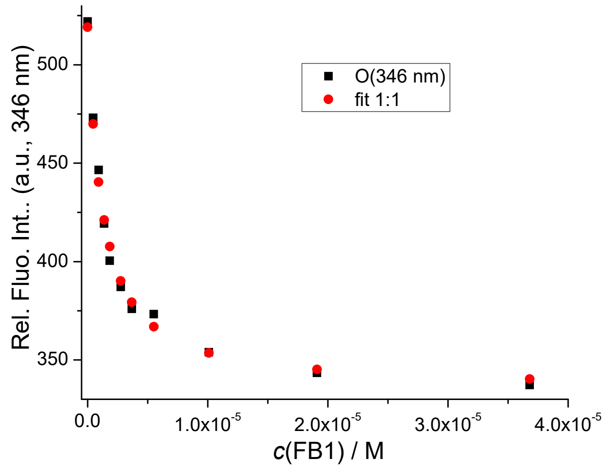

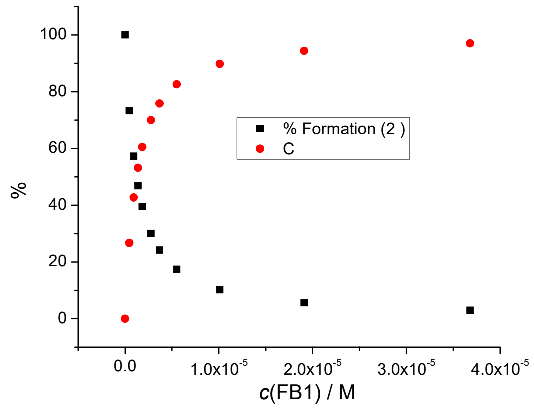

B) **C)**

Figure S4. A) Fluorescent emission spectra of BSA (c(BSA) = 1.86·10^{-7} M in a cuvette) upon titration with $\mathbf{FB_1}$ in water (λ_{exc}= 280 nm, slit: 10 10) at 298 K. Specfit [2] parameters for fluorimetric titration of BSA with FB_1. **B)** Emission dependence of BSA on c($\mathbf{FB_1}$) at λ_{max} = 346 nm (**black** squares – experimental data, **red** diamonds – fit for 1: 1 (BSA: FB_1) stoichiometry). **C)** distribution of spectroscopically active species (**black** squares - free BSA, **red** diamonds - BSA-FB_1 complex in 1:1 stoichiometry).

1. Schneider, C. A., Rasband, W. S., & Eliceiri, K. W. (2012). NIH Image to ImageJ: 25 years of image analysis. *Nature Methods*, *9*(7), 671–675. doi:10.1038/nmeth.2089
2. SPECFIT GLOBAL ANALYSIS, a Program for Fitting, Equilibrium and Kinetic Systems, using Factor Analysis & Marquardt Minimization; Maeder, M., Zuberbuhler, A. D. Nonlinear Least-Squares Fitting of Multivariate Absorption Data, Anal. Chem. 1990, 62, 2220-2224
